# IgG Subclass Profiles of HLA Antibodies Enhance Prediction of C1q-Binding in Kidney Transplant Recipients

**DOI:** 10.3390/diagnostics16020207

**Published:** 2026-01-09

**Authors:** Hyeyoung Lee, Jin Jung, Ae-Ran Choi, Eun-Jee Oh

**Affiliations:** 1 Department of Laboratory Medicine, Korea University Anam Hospital, Korea University College of Medicine, Seoul 02841, Republic of Korea; shomermaid@catholic.ac.kr; 2 Department of Laboratory Medicine, Seoul St. Mary’s Hospital, College of Medicine, The Catholic University of Korea, Seoul 06591, Republic of Korea; jiinj@catholic.ac.kr (J.J.); bibi@cmcnu.or.kr (A.-R.C.); 3 Research and Development Institute for In Vitro Diagnostic Medical Devices, College of Medicine, The Catholic University of Korea, Seoul 06591, Republic of Korea

**Keywords:** IgG subclass, kidney transplantation, HLA, C1q binding

## Abstract

**Background/Objectives**: While standard Luminex single antigen bead (SAB) detects total IgG antibodies, qualitative differences among IgG subclasses may influence their immunologic risk. In particular, complement fixing ability, assessed via C1q binding, is linked to poor transplant outcomes. This study aimed to evaluate the relationship between IgG subclasses and C1q-binding activity in HLA antibodies and to define clinically relevant subclass-specific mean fluorescence intensity (MFI) thresholds for predicting complement binding. **Methods**: We analyzed 4189 HLA IgG bead reactions from sera of 37 kidney transplant recipients using SAB assays for total IgG, IgG1-4 subclasses, and C1q-binding. IgG subclasses were assessed using a modified SAB assay with subclass-specific monoclonal secondary antibodies. **Results**: IgG reactivity (MFI ≥ 1000) was observed in 15.3% of beads (639/4189), with 31.0% (198/639) also positive for C1q binding. IgG^+^C1q^+^ beads exhibited significantly higher MFIs compared with IgG^+^C1q^−^ beads. IgG1 showed positive correlations with both total IgG (*r_s_* = 0.5439, *p* < 0.0001) and C1q MFIs (*r_s_* = 0.4042, *p* < 0.0001), with the strongest correlations at HLA-DQ. Among subclass-positive beads, IgG1 predominated and was strongly associated with C1q binding, whereas isolated IgG2 or IgG4 positivity was rarely C1q-binding. ROC analysis identified an IgG1 MFI threshold of >837 to predict C1q positivity with 73.2% sensitivity and 92.3% specificity, while the cutoff for total IgG MFI was >7881 with 85.4% sensitivity and 88.9% specificity. At the patient level, IgG1-positive immunodominant DSAs were more frequent in antibody-mediated rejection than in non-rejection biopsies **Conclusions**: IgG1 predominates among complement-fixing antibodies and correlates strongly with total IgG and C1q binding. Quantitative IgG subclass assessment, especially IgG1, may serve as a useful predictor of complement activation.

## 1. Introduction

Donor specific HLA antibody (DSA) is associated with antibody mediated rejection (AMR) and graft loss in kidney transplant recipients [1]. Currently, single antigen bead (SAB) assays using Luminex technology have been used to detect total IgG antibody against HLA antigens, providing a semi-quantitative measure of antibody strength through mean fluorescence intensity (MFI) [2]. However, evidence suggests that DSAs vary in their immunologic risk, emphasizing the need to assess qualitative features to improve risk stratification and outcome prediction [3,4,5].

Complement-binding DSAs are strongly associated with AMR and reduced graft survival, highlighting the clinical relevance of complement-activating capacity [6,7,8,9]. C1q and C3d assays allow detection of HLA antibodies capable of initiating the classical complement pathway and provide risk stratification beyond conventional IgG SAB results [10,11]. Loupy et al. reported substantially lower 5-year graft survival among recipients with C1q-positive DSAs, corresponding to a several-fold increase in the risk of graft loss [6]. In addition, the persistence of C1q-positive DSAs after treatment was identified as a strong and independent predictor of inferior graft survival and ongoing antibody-mediated injury in kidney transplant recipients with ABMR [12]. Collectively, these observations highlight the limitations of MFI-based assessment alone and emphasize the need for functional evaluation of DSAs.

IgG subclasses play distinct roles in immune responses and may influence the pathogenicity of DSAs [13]. IgG3 is the most potent activator of complement, followed by IgG1, while IgG2 and IgG4 rarely bind C1q or activate complement [14]. IgG3 and IgG1 also have the highest affinity for the FcgRIIIa activating receptor, mediating NK cell-dependent ADCC [15]. Prior studies have linked IgG1 and IgG3 DSA subclasses to acute rejection, whereas IgG2 and IgG4 are more often associated with subclinical or chronic rejection [3,13,16,17,18,19]. Although subclass profiling of HLA antibodies may have important clinical implications, there is currently no standardized method or widely accepted method for IgG subclass detection in transplantation setting [1]. Previous study has used a modified Luminex SAB assay, in which the conventional pan-IgG secondary antibody is replaced with monoclonal antibodies specific to each IgG subclass (IgG1–IgG4) [20]. However, data on the relationship between IgG subclass distribution and complement-binding activity remain limited.

In this study, we employed a modified Luminex assay to characterize the IgG subclass composition of HLA antibodies in kidney transplant recipients and to evaluate its utility for predicting C1q-binding capacity. By comparing subclass distribution with C1q assay results, we aimed to better define the immunologic features of complement-fixing HLA antibodies and assess the potential value of subclass analysis in improving clinical risk assessment.

## 2. Materials and Methods

### 2.1. Study Design and Samples

This study analyzed remnant serum samples from 37 sensitized kidney transplant recipients with assigned HLA antibody specificities, collected at Seoul St. Mary’s Hospital between March 2018 and September 2019. Patients were included if HLA antibodies were detected in prior SAB screening and adequate serum volume was available. The study comprised 23 men (62.2%) and 14 women (37.8%), with a median age of 46 years (range, 22–64 years). Serum samples were obtained around the time of indication biopsies performed for the evaluation of suspected allograft dysfunction. All serum samples were stored at −70 °C until testing. Only a single freeze–thaw cycle was permitted for all SAB procedures. This study was approved by the Seoul St. Mary’s hospital Institutional Review Board (KC18SESI0856, date of approval: 11 January 2019).

### 2.2. Single Antigen Bead (SAB)-Total IgG Assay

HLA antibodies were detected by LABScreen Single Antigen (One Lambda, Canoga Park, CA, USA) at the time of biopsy. Assay was tested after pretreatment with EDTA as previously described [2]. HLA antibody specificity was interpreted based on baseline mean fluorescence intensity (MFI) values using HLA Fusion™ software (v4.7.1). MFI threshold of >1000 was used to define positivity. The immunodominant DSA of each patient was defined as the DSA with the highest intensity [21].

### 2.3. Single Antigen Bead (SAB)-C1q Assay

The complement-binding activity of anti-HLA antibodies was assessed using the commercial C1qScreen™ assay (One Lambda, Canoga Park, CA, USA). The HLA LABScreen™ and C1qScreen™ platforms enable the detection and characterization of IgG antibodies directed against HLA class I and class II antigens with the ability to bind C1q. These bead-based assays rely on antigen–antibody interactions that are recognized by C1q molecules, and the subsequent binding events are visualized using fluorophore-conjugated detection antibodies. Fluorescence signals were acquired with a dedicated analyzer and interpreted using HLA Fusion™ software version, which assigns antibody specificity by comparing bead fluorescence intensities with the known antigen targets on each bead [7,22]. All procedures were performed according to the instructions provided by the manufacturer. C1q positivity was defined using MFI threshold of 1000.

### 2.4. IgG Subclass Analysis

IgG subclass profiling was conducted using an investigational Luminex-based SAB assay provided by One Lambda. The assay was adapted from the standard SAB protocol, replacing the conventional PE-labeled polyclonal anti-human IgG antibody with PE-conjugated murine monoclonal antibodies specific to each IgG subclass. Millipore. The capture antibodies used were anti-IgG1 (clone HP6001), anti-IgG2 (clone HP6002), anti-IgG3 (clone HP6047), and anti-IgG4 (clone HP6023) (SouthernBiotech, Birmingham, AL, USA). Subclass-specific antibody detection was performed for both HLA Class I and Class II antigens. Antibodies with subclass-specific MFIs above 500 were designated positive according to prior validation studies [16,18,19,23,24,25].

### 2.5. Statistical Analysis

Continuous variables were expressed as mean ± standard deviation or as median with interquartile range, according to distributional characteristics. Group comparisons used Student’s *t*-test or the Mann–Whitney U test, while categorical variables were analyzed with the chi-square test. Correlations among total IgG, IgG subclasses, and C1q MFIs were examined using spearman’s rank correlation coefficient (*r_s_*). Receiver operating characteristic (ROC) curves were used to identify the IgG1 and total IgG MFI cutoff values that best predicted C1q positivity. *p* < 0.05 were considered significant. All analyses were performed with MedCalc^®^ Statistical Software version 23.4.0 (MedCalc Software Ltd., Ostend, Belgium) and GraphPad Prism 10.5.0 for Windows (GraphPad, San Diego, CA, USA).

## 3. Results

### 3.1. Distribution of HLA Antibody Bead Reactions by Total IgG, IgG Subclass and C1q Binding Status

A total of 4189 HLA antibody bead reactions were included, consisting of 1820 (43.5%) HLA class I and 2369 (56.5%) HLA class II beads (Table 1). Of all bead reactions, 15.3% (639/4189) were positive for total IgG antibodies (MFI ≥ 1000), with the highest positivity observed for HLA-DQ (41.3%), followed by HLA-B (22.4%) and HLA-DR (17.4%). Median total IgG MFI values varied markedly by HLA locus, ranging from 2441 for HLA-A to 17,672 for HLA-C. Of the IgG-positive beads, 198 (4.7% of all beads; 31.0% of IgG-positive beads) were also positive for C1q binding, most abundantly within HLA-DQ (54.5%), followed by HLA-DR (19.7%) and HLA-B (15.7%). In contrast, no HLA-DP bead exhibited C1q positivity. Median MFIs for C1q-positive beads exceeded those for C1q-negative beads across nearly all loci, with the highest values noted among HLA-DR and HLA-DQ beads.

**Figure 1:**
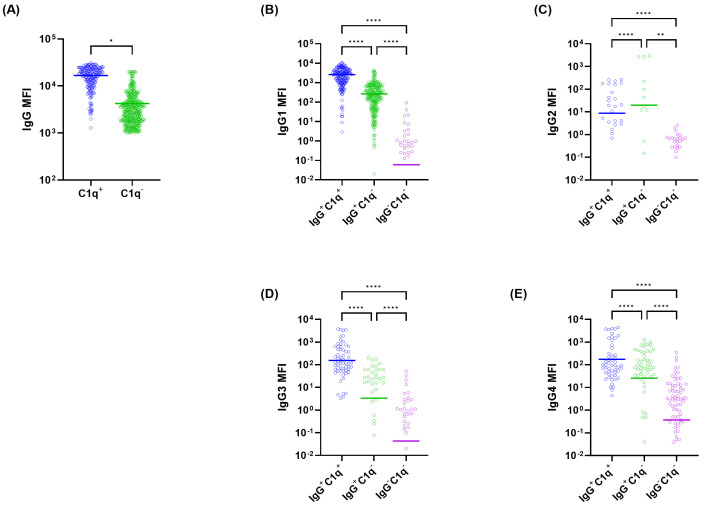
Distribution of mean fluorescence intensity (MFI) levels for total IgG (**A**) and IgG subclasses (**B**–**E**) in kidney transplant recipient sera classified by IgG and C1q-binding status. (**A**) MFI levels of total IgG compared between C1q-positive (C1q^+^) and C1q-negative (C1q^−^) groups. (**B**–**E**) MFI levels for IgG1 (**B**), IgG2 (**C**), IgG3 (**D**), and IgG4 (**E**) are shown according to antibody profiles: IgG^+^/C1q^+^, IgG^+^/C1q^−^, and IgG^−^/C1q^−^. Statistical significance is denoted by asterisks (* *p* < 0.05, ** *p* < 0.01, **** *p* < 0.0001).

Figure 1 compares the MFI distributions of total IgG and each IgG subclass between IgG^+^C1q^+^ (*n*=198) and IgG^+^C1q^−^ (*n* = 441) bead groups. Compared with IgG^+^C1q^−^ beads, IgG^+^C1q^+^ beads demonstrated significantly higher MFIs for total IgG, IgG1, IgG3, and IgG4 (all *p* < 0.0001). The median total IgG MFI values [min–max] for IgG^+^C1q^+^ and IgG^+^C1q^−^ beads were 17,660 [1288–31,091] and 2922 [1011–20,530], respectively. The median IgG1 MFI was also markedly higher in C1q-positive than in C1q-negative beads (2082 vs. 19, *p* < 0.0001), with comparable differences observed for IgG3 and IgG4 (both *p* < 0.0001). In contrast, IgG2 MFIs were significantly higher in IgG^+^C1q^−^ beads (*p* < 0.0001), showing an inverse pattern relative to other subclasses.

### 3.2. Correlation of IgG Subclass MFI with Total IgG and C1q Reactivity by HLA Locus

**Figure 2:**
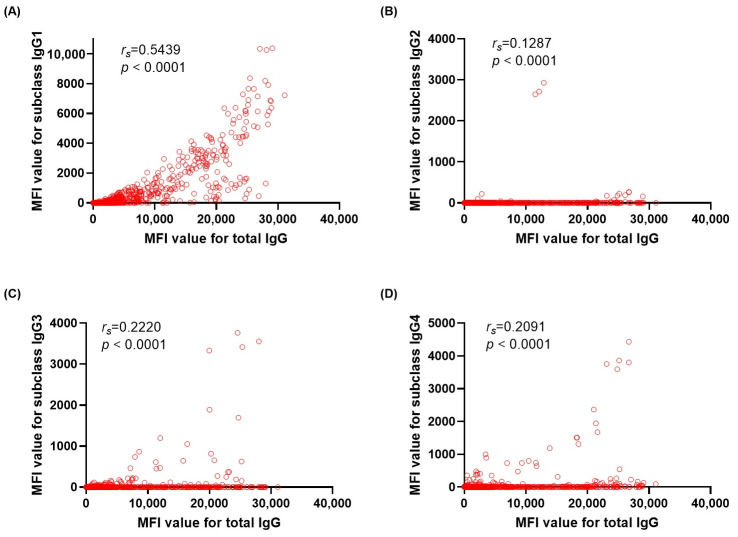
Correlation between total IgG MFI and IgG subclasses. Correlations between total IgG mean fluorescence intensity (MFI) and subclass-specific MFI for IgG1 (**A**), IgG2 (**B**), IgG3 (**C**), and IgG4 (**D**) MFIs, respectively. Spearman correlation coefficients (*r_s_*) and associated *p*-values are presented within each panel.

**Figure 3:**
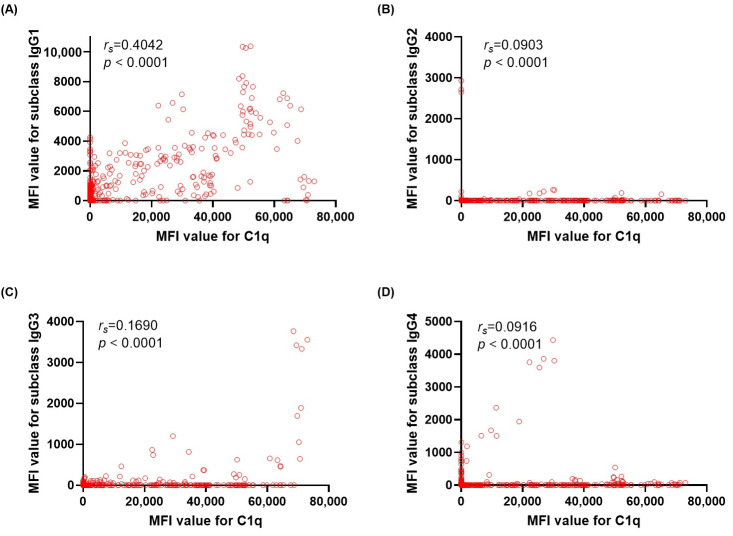
Correlation between C1q MFI and IgG subclasses. Correlations between C1q mean fluorescence intensity (MFI) and subclass-specific MFI for IgG1 (**A**), IgG2 (**B**), IgG3 (**C**), and IgG4 (**D**) MFIs, respectively. Spearman correlation coefficients (*r_s_*) and associated *p*-values are presented within each panel.

**Figure 4:**
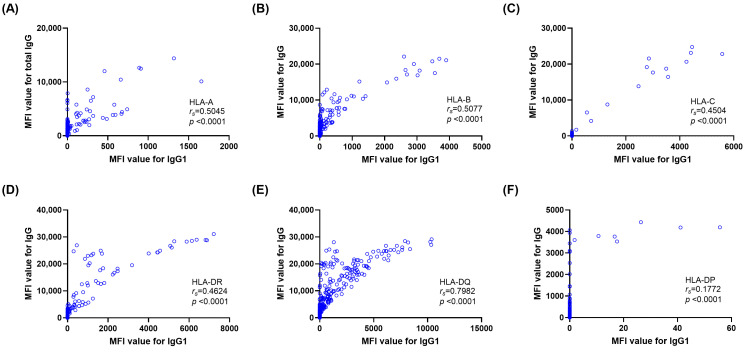
Correlations between total IgG mean fluorescence intensity (MFI) and IgG1 subclass MFI values across HLA loci. Six scatterplots show correlations across HLA-A (**A**), -B (**B**), -C (**C**), -DR (**D**), -DQ (**E**), and -DP (**F**) bead groups. Spearman correlation coefficients (*r_s_*) and corresponding *p*-values are shown in each panel.

**Figure 5:**
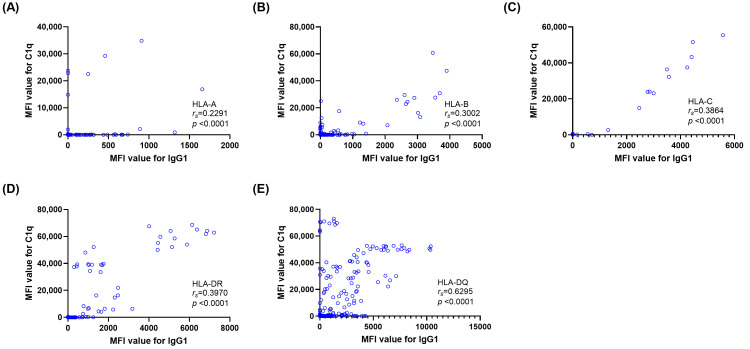
Correlations between C1q mean fluorescence intensity (MFI) and IgG1 subclass MFI values across HLA loci. Scatterplots show correlations across HLA-A (**A**), -B (**B**), -C (**C**), -DR (**D**), and -DQ (**E**) bead groups. Spearman correlation coefficients (*r_s_*) and corresponding *p*-values are shown in each panel.

To evaluate the contribution of each IgG subclass to overall antibody reactivity and complement-binding capacity, we assessed the correlations of subclass-specific MFI values and total IgG or C1q MFI values. Among subclass beads, IgG1 MFI showed strong correlation with total IgG MFI (*r_s_*= 0.5439, *p* < 0.0001), with weak to moderate correlation for IgG2 (*r_s_* = 0.1287, *p* <0.0001), IgG3 (*r_s_* = 0.2220, *p* <0.0001), and IgG4 (*r_s_* = 0.2091, *p* < 0.0001) (Figure 2). For C1q-positive beads, IgG1 also showed the strongest correlation with C1q (*r_s_* = 0.4042, *p* <0.0001), while with weak correlation for IgG2 (*r_s_* = 0.0903, *p* <0.0001), IgG3 (*r_s_* = 0.1690, *p* <0.0001), and IgG4 (*r_s_* = 0.0916, *p* <0.0001) (Figure 3). Correlation analyses between IgG1 MFI and total IgG MFI levels by HLA locus showed consistently positive relations across HLA loci (Figure 4). Correlations between IgG1 and total IgG MFI were as follows: HLA-DQ (*r_s_* = 0.7982, *p* < 0.0001), HLA-B (*r_s_* = 0.5077, *p* < 0.0001), HLA-A (*r_s_* = 0.5045, *p* < 0.0001), HLA-DR (*r_s_* = 0.4624, *p* < 0.0001), and HLA-C (*r_s_* = 0.4504, *p* < 0.0001), with the weakest at HLA-DP (*r_s_* = 0.1772, *p* < 0.0001). Similar correlation trends were observed for IgG1 and C1q MFI levels. Correlations between IgG1 and C1q MFI were HLA-DQ (*r_s_* = 0.6295, *p* < 0.0001), HLA-DR (*r_s_* = 0.3970, *p* < 0.0001), HLA-C (*r_s_* = 0.3864, *p* < 0.0001), HLA-B (*r_s_* = 0.3002, *p* < 0.0001), and HLA-A (*r_s_* = 0.2291, *p* < 0.0001), with no correlation with HLA-DP (Figure 5).

### 3.3. IgG Subclass Positivity in Relation to Total IgG and C1q Binding Activity

Of the 639 total IgG-positive beads, 242 (37.9%) exhibited positive for at least one IgG subclass using an MFI cutoff of ≥500 (Table 2). Among these IgG subclass-positive beads, the majority involved IgG1, either alone or in combination with other subclass beads (*n* = 227, 93.8%). Among subclass-positive beads, 66.5% (161/242) were also positive for C1q binding. Notably, all IgG3-positive beads (15/15) were C1q-positive, whereas 67.4% (153/227) of IgG1 containing beads showed C1q positivity. Specifically, beads positive for IgG1-alone showed 65.4% (134/205) C1q positivity, compared to 20.0% (1/5) for IgG4-alone beads; none of the IgG2-only positive beads (*n* = 3) were C1q-positive.

**Figure 6:**
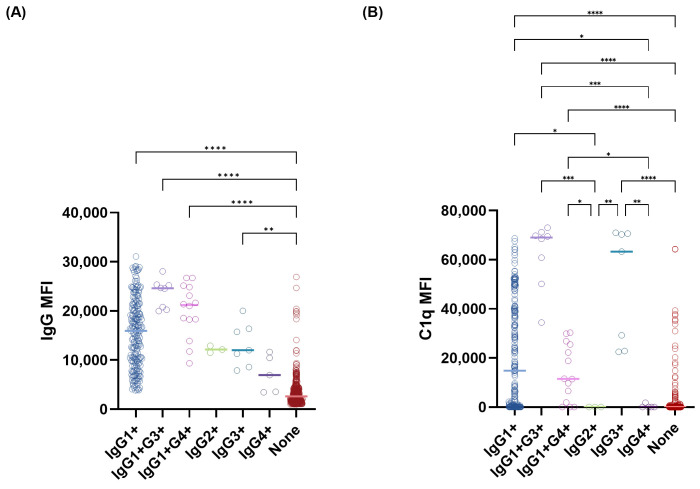
Total IgG MFI and C1q MFI distributions according to IgG subclass patterns. Plots illustrate total IgG MFI (**A**) and C1q MFI (**B**) distributions according to IgG subclass patterns, including IgG1-only, IgG2-only, IgG3-only, IgG4-only, multi-subclass combinations, and subclass-negative beads. Statistical significance is denoted by asterisks (* *p* < 0.05, ** *p* < 0.01, *** *p* < 0.001, **** *p* < 0.0001).

Total IgG MFI values were compared across IgG subclass patterns (Figure 6). Although, there was no statistically significant differences, beads positive for IgG1+G3+ and IgG1+G4+ demonstrated substantially higher total IgG MFIs (median [min–max], 24,628 [19,970–28,044] and 21,201 [9349–26,716], respectively). In contrast, IgG3 single-positive beads showed only intermediate total IgG intensity (median 12,004 [7900–20,002]), while IgG4+ and IgG2+ beads exhibited low reactivity. The subclass-negative group showed the lowest signal levels overall. A similar distributional trend was observed for C1q-binding MFIs. IgG1+G3+ and IgG3+ beads demonstrated the strongest C1q reactivity (median [min–max], 69,006 [34,420–73,037] and 63,302 [22,507–70,948], respectively), followed by IgG1 and IgG1+G4+, whereas IgG2+, IgG4+, and subclass-negative groups showed negligible C1q activation. Notably, the IgG1+G4+ group demonstrated significantly higher C1q MFI compared with the IgG4-only group (11,480 [13–30,322] vs. 0 [0–1771], *p* <0.05).

### 3.4. Predictive Performance of Total IgG and IgG Subclasses for C1q Binding Activity

**Figure 7:**
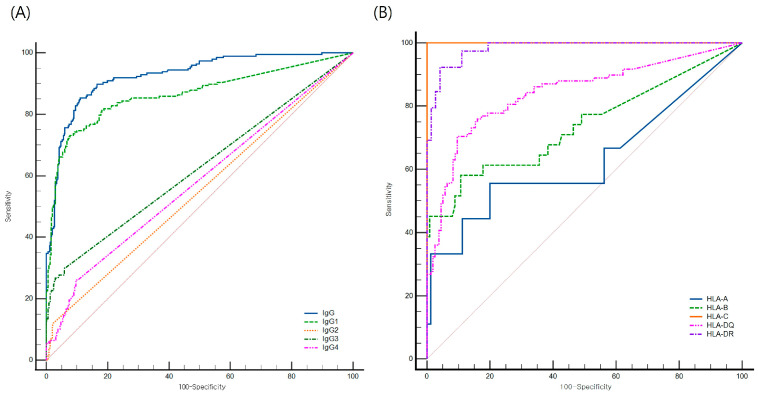
Receiver operating characteristic (ROC) curve analysis for prediction of C1q binding by total IgG and IgG subclasses. (**A**) ROC curves showing predictive performance of total IgG and IgG subclasses for C1q-binding positivity. Total IgG MFI yielded the highest AUC (0.927), followed by IgG1 (0.865). (**B**) Subanalysis by HLA locus showing highest predictive accuracy for IgG1 at HLA-C (AUC = 1.0) and HLA-DR (0.983). The red diagonal line indicates the line of no discrimination (AUC = 0.5).

To evaluate the predictive performance of total IgG and IgG subclasses for C1q-binding activity, ROC curve analysis was performed (Figure 7). Among 639 total IgG-positive beads (198 C1q-positive, 441 C1q-negative), total IgG revealed the best discrimination with an optimal cutoff > 7881, yielding 85.4% sensitivity and 88.9% specificity (AUC = 0.927, 95% CI: 0.904–0.946). The IgG1 subclass MFI also showed good predictive ability, with a cutoff > 837 yielding 73.2% sensitivity and 92.3% specificity (AUC = 0.865, 95% CI: 0.836–0.891). In contrast, IgG2, IgG3, and IgG4 yielded lower AUCs (0.549, 0.625, and 0.580, respectively). When stratified by HLA locus, IgG1 MFI showed perfect predictive capacity at HLA-C (AUC = 1.0), with 100% sensitivity and specificity at a cutoff of ≥731. HLA-DR followed closely (AUC = 0.983), achieving 92.3% sensitivity and 95.8% specificity at ≥716. HLA-DQ showed moderate predictive performance (AUC = 0.842), with 70.4% sensitivity and 90.4% specificity at a cutoff of ≥1187.

### 3.5. Patient-Level Characteristics of Immunodominant DSA According to Biopsy Diagnosis and Pre-Transplant DSA Status

Patient-level characteristics of immunodominant DSAs were compared between patients with AMR (*n* = 27) and those without AMR (*n* = 10), the latter including cases of T cell–mediated rejection, acute tubular necrosis, myocardial infarction, BK virus nephropathy, and interstitial fibrosis/tubular atrophy (Table 3). The median immunodominant DSA MFI was higher in the AMR group than in the non-AMR group (9938 [IQR, 4875–19,766] vs. 3879 [IQR, 3402–12,004]), although this difference did not reach statistical significance (*p* = 0.0873). C1q-positive immunodominant DSAs were detected in 11 patients (40.7%) with AMR and in 3 patients (30.0%) without AMR (*p* = 0.5566). In contrast, IgG1-positive immunodominant DSAs were significantly more frequent among patients with AMR compared with those without AMR (66.7% vs. 30.0%, *p* = 0.0484). The proportion of immunodominant DSAs that were both IgG1-positive and C1q-positive did not differ significantly between the AMR and non-AMR groups (33.3% vs. 30.0%, *p* = 0.8510).

Subgroup analysis according to pre-transplant DSA status revealed distinct patterns. Among patients with pre-transplant DSA (*n* = 8), 6 (75.0%) developed biopsy-proven AMR. Within this subgroup, IgG1-positive immunodominant DSAs were present in 4 of the 6 AMR cases, whereas none exhibited C1q-positive immunodominant DSAs, indicating the absence of detectable complement-binding activity despite prior sensitization. In contrast, among patients without pre-transplant DSA (*n* = 29), 21 (72.4%) developed AMR. In this group, IgG1-positive immunodominant DSAs were observed in 14 patients, and C1q-positive immunodominant DSAs in 11 patients, demonstrating the emergence of functionally active, complement-binding antibodies de novo after transplantation. It should be noted that IgG subclass and C1q analyses were performed only on post-transplant biopsy-era sera, as pre-transplant samples were not available for functional profiling Overall, these findings indicate that pre-transplant DSA status alone did not reliably predict IgG subclass composition or complement-binding capacity at the time of biopsy. This highlights the substantial heterogeneity in functional antibody profiles and demonstrates the importance of post-transplant functional antibody characterization

## 4. Discussion

This study provides a comprehensive evaluation of HLA-specific IgG subclass profiles and C1q-binding activity in kidney transplant recipients. Our findings reinforce the immunologic heterogeneity of HLA antibodies and offer valuable insights into their functional relevance in complement activation.

Although literature on IgG subclass roles in solid organ transplantation remains limited and heterogeneous regarding study design, patient selection, and outcome measures, the predominance of IgG1 subclass was consistently observed [16,18,20,23,24,26,27,28,29]. Our findings align with this consensus, confirming IgG1 as the most prevalent subclass, despite variable proportions of other subclasses across studies.

IgG3, known for its potent complement-fixing ability owing to its extended hinge region and highest affinity for C1q, was infrequently detected in this study. This observation is consistent with a previous study by Navas et al. [24]. The low detectability of IgG3 may be explained by its early expression during class-switch recombination and relatively short half-life in circulation, both of which may reduce its presence in peripheral blood and limit its detection by standard solid-phase assays [30,31].

Among IgG1-positive beads, a significant subset (33.8%) lacked C1q-binding capacity, highlighting that subclass presence alone is insufficient to predict complement activation. Previous reports similarly indicated that C1q positivity occurs predominantly in sera with high MFI IgG1 or the presence of all four subclasses [20]. Schaub et al. demonstrated that C1q binding correlates with antibody density, where many antibodies bearing complement-binding subclasses fail to induce C1q binding in vitro [32].

Notably, IgG1 MFI responses varied across HLA loci, with the highest levels at HLA-DQ suggesting that DSA specificity and subclass distribution jointly influence the risk of complement-mediated injury. HLA-DQ is especially important due to its immunogenicity and strong association with chronic AMR and graft loss [33,34]. These findings underscore the clinical importance of detailed subclass profiling in post-transplant humoral response evaluation.

A major limitation in the wider adoption of IgG subclass analysis remains the lack of standardized, commercially available assays and universal cutoffs. As a result, there is no universally accepted cut-off value for each subclass, and previous studies have used a variety of criteria, including fixed MFI thresholds as 500 [16,18,19,23,24,25,35,36,37], negative control-based calculations [38], and proportional adjustments [39]. Recent advancements, such as the development of novel flow cytometric methods enabling direct detection of recipient IgG subclass antibodies binding to donor cells, offer promising avenues for accurate subclass-specific analysis in a donor-specific context [40].

Importantly, our ROC curve analysis demonstrated that IgG1 MFI is a robust predictor of C1q positivity, with high sensitivity and specificity at optimized thresholds. This finding highlights the clinical utility of subclass-specific quantification, particularly for IgG1, in identifying patients at increased risk for complement-mediated antibody injury. However, total IgG exhibited an even higher AUC for predicting C1q binding, demonstrating the limitations of using subclass data alone—an observation consistent with a previous study [41].

The patient-level analyses based on immunodominant donor-specific antibodies indicate that IgG subclass composition, particularly IgG1, is associated with biopsy-proven AMR, whereas immunodominant DSA MFI and C1q-binding positivity were not. These findings suggest that functional characteristics of DSAs may provide clinically relevant information beyond conventional quantitative or complement-binding metrics. In addition, the heterogeneous IgG subclass and C1q-binding profiles observed irrespective of pre-transplant DSA status underscore the dynamic nature of humoral alloimmune responses and highlight the potential value of functional antibody profiling in interpretating biopsy findings.

The present study has several limitations. The modest sample size from a single center may restrict generalizability, and the IgG subclass assay used was a modified research-use-only platform without inter-laboratory standardization. In addition, the lack of longitudinal clinical outcome data precluded assessment of long-term graft function and survival. Pre-transplant sera were not available for subclass or C1q analysis, preventing longitudinal evaluation of antibody functional evolution. Detailed information regarding individual sensitizing events was also incomplete due to the retrospective study design, limiting stratified analyses by sensitization pathway.

Future prospective studies incorporating serial immunologic assessment with clinical outcomes, including biopsy-proven rejection, graft function, and survival, are needed to validate these findings. Furthermore, systematic documentation of sensitizing histories and temporal analysis of antibody functional changes will be essential to clarify how distinct sensitization pathways influence IgG subclass distribution and complement-binding capacity.

In conclusion, our study highlights the value of integrating IgG subclass analysis with C1q-binding assays in assessing HLA antibodies. IgG1 emerges as the dominant subclass linked to complement-fixing activity, offering improved granularity beyond total IgG quantification. These findings pave the way for functional antibody profiling to enhance risk assessment and ultimately improve transplant outcomes.

## 5. Conclusions

This study demonstrates that IgG subclass profiling provides important qualitative insights into the heterogeneity of HLA antibodies and their complement-fixing potential in kidney transplant recipients. IgG1 emerged as the dominant subclass associated with high total IgG burden and C1q-binding activity, and quantitative subclass assessment—particularly IgG1 MFI—enhanced the prediction of complement activation beyond standard total IgG SAB results. These findings underscore the potential value of integrating functional antibody characterization into post-transplant immunologic risk assessment.

## Figures and Tables

**Table 1:** Distribution of HLA antibody bead reactions by total IgG, and C1q binding status.

		Total	HLA Class I			HLA Class II		
			A	B	C	DR	DQ	DP
Total bead numbers		4189	589 (14.0%)	950 (22.7%)	281 (6.7%)	900 (21.5%)	694 (16.6%)	775 (18.5%)
Total IgG positivity	bead, no (%)	639	89 (13.9%)	143 (22.4%)	15 (2.3%)	111 (17.4%)	264 (41.3%)	17 (2.7%)
	MFI, Median (min-max)	4236 (1011–31,091)	2441 (1012–14,391)	3566 (1053–22,111)	17,672 (1357–24,762)	4250 (1011–31,091)	7810 (1029–29,118)	3534 (1064–4431)
Total IgG+C1q+	bead, no (%)	198	9 (4.5%)	31 (15.7%)	11 (5.6%)	39 (19.7%)	108 (54.5%)	0
	MFI, Median (min-max)	17,660 (1288–731,091)	8589 (1282–12,645)	9450 (2630–22,111)	19,165 (8772–24,762)	23,013 (5691–31,091)	18,226 (2521–29,118)	0
Total IgG+C1q-	bead, no (%)	441	80 (18.1%)	112 (25.4%)	4 (0.9%)	72 (16.3%)	156 (35.4%)	17 (3.9%)
	MFI, Median (min-max)	2922 (1011–20,530)	2226 (1012–14,391)	2869 (1053–12,909)	2956 (1357–6544)	2324 (1011–12,455)	3903 (1029–20,530)	3534 (1064–4431)

Abbreviations: MFI, mean fluorescence intensity; min, minimum; max, maximum.

**Table 2:** IgG subclass and C1q profiles of IgG positive beads (*n* = 639).

IgG Subclass	Total (*n* = 639)	%	C1q Negative (*n* = 441)	%	C1q Positive (*n* = 198)	%	*p* Value
None subclass detected, *n* (%)	397	62.1	360	90.7	37	9.3	<0.0001
Any subclass detected, *n* (%)	242	37.9	81	33.5	161	66.5	
IgG subclass pattern, *n* (%)							
G1 alone	205	84.7	71	34.6	134	65.4	<0.0001
G1+G3	7	2.9	0	0.0	7	100.0	0.0004
G1+G3+G4	1	0.4	0	0.0	1	100.0	NS
G1+G4	14	5.8	3	21.4	11	78.6	0.0003
G2 alone	3	1.2	3	100.0	0	0.0	NS
G3 alone	7	2.9	0	0.0	7	100.0	0.0004
G4 alone	5	2.1	4	80.0	1	20.0	NS

Abbreviations: NS, non-significant.

**Table 3:** Patient-level characteristics of immunodominant DSA according to biopsy diagnosis.

Immunodoninant DSA Characteristics	AMR (*n* = 27)	Non-AMR (*n* = 10) *	*p* Value
MFI, median (IQR)	9938 (4875–19,766)	3879 (3402–12,004)	0.0873
C1q positivity, *n* (%)	11 (40.7%)	3 (30.0%)	0.5566
IgG1 positivity, *n* (%)	18 (66.7%)	3 (30.0%)	0.0484
IgG1 and C1q positivity, *n* (%)	9 (33.3%)	3 (30.0%)	0.8510

Abbreviations: MFI, mean fluorescence intensity; AMR, antibody mediated rejection, IQR Interquartile Range. * Non-AMR includes T cell–mediated rejection, acute tubular necrosis, microinflammation, BK virus nephropathy, and interstitial fibrosis/tubular atrophy.

## Data Availability

The data presented in this study are available on request from the corresponding author, subject to ethical and institutional restrictions.

## Abbreviations

The following abbreviations are used in this manuscript:

SAB	Single antigen bead
MFI	Mean fluorescence intensity
DSA	Donor specific HLA antibody
HLA	Human Leukocyte Antigen
